# Nucleotide-binding sites can enhance *N*-acylation of nearby protein lysine residues

**DOI:** 10.1038/s41598-020-77261-1

**Published:** 2020-11-20

**Authors:** Andrew M. James, Anthony C. Smith, Shujing Ding, Jack W. Houghton, Alan J. Robinson, Robin Antrobus, Ian M. Fearnley, Michael P. Murphy

**Affiliations:** 1 Medical Research Council Mitochondrial Biology Unit, University of Cambridge, Cambridge, CB2 0XY UK; 2 Cambridge Institute of Medical Research, University of Cambridge, Cambridge, CB2 0XY UK; 3 Department of Medicine, University of Cambridge, Cambridge, CB2 0QQ UK

**Keywords:** Biochemistry, Chemical biology

## Abstract

Acyl-CoAs are reactive metabolites that can non-enzymatically *S-*acylate and *N*-acylate protein cysteine and lysine residues, respectively. *N-*acylation is irreversible and enhanced if a nearby cysteine residue undergoes an initial reversible *S-*acylation, as proximity leads to rapid *S* → *N*-transfer of the acyl moiety. We reasoned that protein-bound acyl-CoA could also facilitate *S* → *N*-transfer of acyl groups to proximal lysine residues. Furthermore, as CoA contains an ADP backbone this may extend beyond CoA-binding sites and include abundant Rossmann-fold motifs that bind the ADP moiety of NADH, NADPH, FADH and ATP. Here, we show that excess nucleotides decrease protein lysine *N-*acetylation in vitro. Furthermore, by generating modelled structures of proteins *N*-acetylated in mouse liver, we show that proximity to a nucleotide-binding site increases the risk of *N-*acetylation and identify where nucleotide binding could enhance *N-*acylation in vivo. Finally, using glutamate dehydrogenase as a case study, we observe increased in vitro lysine *N-*malonylation by malonyl-CoA near nucleotide-binding sites which overlaps with in vivo* N-*acetylation and *N-*succinylation. Furthermore, excess NADPH, GTP and ADP greatly diminish *N-*malonylation near their nucleotide-binding sites, but not at distant lysine residues. Thus, lysine *N-*acylation by acyl-CoAs is enhanced by nucleotide-binding sites and may contribute to higher stoichiometry protein *N-*acylation in vivo*.*

## Introduction

Acyl-CoAs are central metabolites in the oxidation of carbohydrate and fat in the mitochondrial matrix, and provide the building blocks for biosynthetic reactions in the cytosol^1^. However, these metabolites also impact cellular function by *N*-acylating the ε-amino group of protein lysines. The biological significance of this *N*-acylation is implied by the existence of several sirtuins (Sirt1-7), which use NAD^+^ to remove acyl groups from protein lysines, and their association with a wide range of degenerative diseases including cancer, ageing and diabetes^2,3^. As *N-*acylation displays some site-specificity it was initially considered as a regulatory modification, allowing the cell to respond to changes in acyl-CoA, the acyl-CoA/CoA ratio or NAD^+^. However, some reassessment was required after observing several thousand sites of lysine *N-*acetylation in vivo^4–6^, the vast majority of which have a very low (~ 0.1%) stoichiometry of acetylation^5,7–9^, and that many other *N-*linked acyl modifications on lysine residues are generated in vivo without known transferase enzymes^10–12^. This was reinforced by the realisation that protein lysines can be non-enzymatically *N*-acylated by acyl-CoAs^7,13,14^. Consequently, it was proposed that acyl-CoAs represent a ‘carbon stress’, through which chronic exposure to acyl-CoAs causes cumulative cellular damage contributing to degenerative diseases and ageing, perhaps explaining the beneficial effects of sirtuins and dietary restriction^8,15–17^. Supporting this proposal, an analysis of sites of *N-*acetylation in vivo showed they are significantly less conserved than non-acetylated sites within vertebrate genomes^14^.

**Figure 1:**
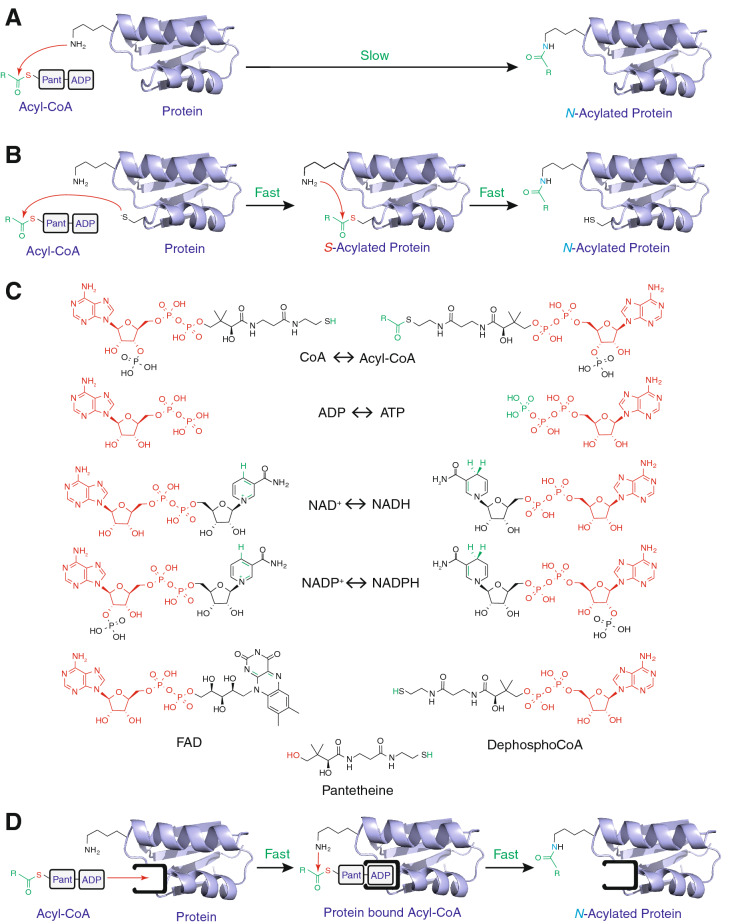
Nucleotide-binding sites could facilitate *N-*acylation of proximal lysine residues by acyl-CoAs. (**A**) The thioester of an acyl-CoA can directly and irreversibly *N-*acylate a lysine residue, but the high pK_a_ of a primary amine (~ 10.5) means this reaction is relatively slow^13^. (**B**) In contrast, the reaction of an acyl-CoA with a protein cysteine thiolate (pK_a_ =  ~ 8.5) is ~ 100-fold faster, but reversible. However, once the thioester localises to the protein surface it can react quickly and irreversibly with nearby lysine amines^7,9,14,17^. (**C**) Many common metabolic cofactors share an ADP-moiety with CoA. (**D**) ADP-binding sites could also localise acyl-CoAs to the protein surface causing rapid and irreversible *N-*acylation of nearby primary amines. *Pant* pantetheine.

Nevertheless, specific lysine residues or proteins are targeted for *N-*acylation and this can create the impression that *N-*acylation is regulatory. However, while some lysine residues may be intentionally targeted, enhanced *N-*acylation on its own is not necessarily proof of biological regulation. This is because non-enzymatic *N-*acylation occurs when the amine group (pK_a_ ~ 10.5) of a lysine residue deprotonates to become a nucleophile and attacks the thioester carbonyl of an acyl-CoA to generate a stable amide-linked protein modification (Fig. 1A)^13^. Enhanced *N-*acylation of certain lysine residues can occur if their pK_a_ is lower or if the protein acts as a scaffold to increase the local thioester concentration near them (Fig. 1B)^7,14,17,18^. One example of the later is when *S-*acylation of proximal cysteine residues catalyses *N*-acylation of nearby lysines^7,9,14,19^. This reaction is analogous to ubiquitination where E3 ubiquitin ligases act as adaptors to localise *S-*ubiquitinated E2 proteins close to proteins destined to be *N-*ubiquitinated. In both cases this *S → S → N*-transfer strategy is effective because proximity enhances the second rate-limiting step. Interestingly, CoA shares an ADP backbone with many nucleotide cofactors such as ATP, NADH, NADPH and FADH (Fig. 1C) and this common backbone is recognised and bound by the ubiquitous Rossmann fold motif^20^. Thus, we reasoned that proteins with nucleotide binding sites could act as scaffolds to enhance *N-*acylation of nearby lysine residues by acyl-CoAs (Fig. 1D).

Here we show that lysine *N-*acylation by acyl-CoAs is enhanced by interactions between acyl-CoAs and nucleotide-binding sites on the protein surface, and that this mechanism may contribute to elevated protein *N-*acylation in vivo.

## Results

### Excess adenine nucleotides decrease lysine *N*-acetylation of mitochondrial proteins by acetyl-CoA in vitro

**Figure 2:**
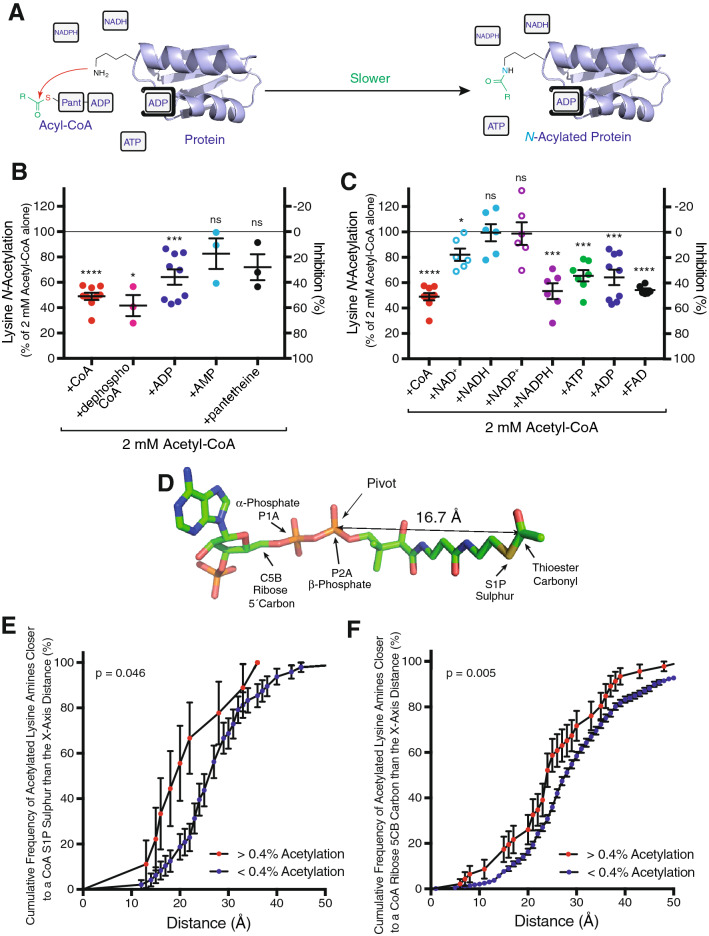
*N-*acetylation of protein by acetyl-CoA is facilitated by nucleotide-binding sites. (**A**) Acetyl-CoA associates with nucleotide binding sites leading to faster protein *N-*acetylation and this is slowed by excess adenine nucleotides competing for these sites. (**B**,**C**) Excess adenine nucleotides decrease protein *N-*acetylation. Bovine mitochondrial membrane proteins were incubated with 2 mM acetyl-CoA for 6 h at 37 °C with or without 10 mM excess adenine nucleotides. *N-*acetylated proteins were separated by SDS-PAGE and quantified by western blot using an anti-acetyllysine antibody. After subtraction of the no acetyl-CoA signal from all lanes, data was expressed relative to the signal from proteins incubated with 2 mM acetyl-CoA alone. Data were the mean ± SEM (n = 3–9). Significance was determined using a paired two-tailed t-test relative to the 2 mM acetyl-CoA alone signal from the same western blot; *, p < 0.05; **, p < 0.01; ***, p < 0.001; ****, p < 0.0001. (**D**) Atoms of acetyl-CoA used for modelling in Supplementary Table S1. The distance from the pivot β-phosphate (2PA) of the bound ADP moiety recognized by a Rossmann fold to the tethered reactive thioester carbonyl is shown. (**E**,**F**) Increased lysine *N-*acetylation occurred near nucleotide binding sites in mouse liver in vivo. *N-*acetylated lysine residues were grouped by their stoichiometry of *N-*acetylation^5^ and ranked by the distance from NZ amine of *N-*acetylated lysine residues to either the thioester sulphur (S1P; **E**) or ribose 5′ carbon (5CB; **F**) atoms of protein-bound nucleotides (Supplementary Table S1). Data are presented as Kaplan–Meier plots of cumulative frequency as a function of separation distance ± SE with statistical significance determined using a Gehan–Breslow–Wilcoxon test.

Previously we had observed a high apparent *K*_*M*_ of ~ 5 mM for *N-*acetylation of mitochondrial membrane proteins by acetyl-CoA in vitro and that this *N-*acetylation was sensitive to CoA^7^. This could occur if acetyl-CoA binds weakly to the surface of proteins before transferring its acetyl moiety. We reasoned that such a CoA-protein association would enhance *N-*acetylation of nearby lysine residues (Fig. 1D) via a mechanism that could be antagonised by co-incubation with structural mimetics of CoA (Fig. 2A). To assess this possibility, fragments of bovine heart mitochondrial membranes were incubated with 2 mM acetyl-CoA and the effect of 10 mM CoA on the degree of protein lysine *N-*acetylation was quantified (Fig. 2B). This showed that 51 ± 3% of *N-*acetylation by acetyl-CoA was prevented by a fivefold excess of CoA (Fig. 2C). A similar magnitude effect was observed with 10 mM dephosphoCoA (Fig. 2B). As these effects could be explained thermodynamically by the effect of the free thiols of CoA or dephosphoCoA on the acetylation potential of acetyl-CoA we next assessed the effect of the component parts of acetyl-CoA (10 mM) on lysine *N-*acetylation. Adding ADP prevented 36 ± 6% of the acetylation, whereas adding AMP was less effective at suppressing acetylation (Fig. 2B). Adding pantetheine, the non-nucleotide thiol-containing component of CoA, was also less effective (Fig. 2B). Thus, association of the nucleotide diphosphate moiety of acetyl-CoA with protein appears to enhance lysine *N-*acetylation.

This mechanism of *N-*acylation may not be limited to proteins where CoA is a high-affinity substrate. CoA, NADPH, NADH, ATP and FADH all share an ADP backbone (Fig. 1C), and binding sites for these cofactors are all based around the Rossmann-fold structural motif that recognises ADP^21^. Consequently, any protein containing an ADP-binding Rossmann fold could potentially bind acyl-CoAs, leading to enhanced *N-*acylation of nearby lysine residues through this association^22^. When fragments of bovine heart mitochondrial membranes were incubated with 2 mM acetyl-CoA and 10 mM of various nucleotides, the metabolic cofactors ATP, NADPH and FAD, all significantly decreased protein lysine *N-*acetylation (Fig. 2C).

This result suggests the nucleotide moiety of acetyl-CoA can cause its association with the surface of proteins that bind common nucleotide-based cofactors, leading to enhanced *N-*acetylation of nearby lysine residues.

### A 3D structural library of mouse liver proteins with bound nucleotides and *N*-acetylated lysines

Although the above data were consistent with our initial hypothesis, the presence of other competing non-enzymatic mechanisms for *N-*acylation (Fig. 1A,B) made definitive analysis difficult. Therefore, to explore whether nucleotide-binding sites can enhance *N-*acylation and identify candidate proteins for further detailed investigation, we generated a dataset of modelled *N-*acetylated protein structures with bound ADP-based cofactors and related artificial structural analogues (Supplementary Table S1). We began with an in vivo dataset of 4319 *N-*acetylated lysine residues in mouse liver^5,6^. From this dataset we took the protein sequences of the *N-*acetylated peptides and, using the most homologous existing protein structure as a template, we built single-subunit models of the mouse proteins with their bound nucleotide analogues^14,23^. Then we calculated the distances from the amine nitrogen atom (NZ) of each protein lysine to the bound nucleotide ligand atom equivalent to the thiol sulphur atom (S1P), the β-phosphate (P2A), α-phosphate (P1A) and the ribose 5′ carbon (C5B) of acetyl-CoA (Fig. 2D). Analysis of the small subset of proteins where the bound analogue contains an atom equivalent to the S1P of CoA indicated that lysine residues with higher *N-*acetylation stoichiometry (> 0.4%, n = 9) were significantly more likely to be near this S1P atom of the bound CoA analogue than less *N-*acetylated residues (< 0.4%, n = 48; Fig. 2E and Supplementary Table S1).

However, assessing proteins that bind cofactors such as ADP, NAD(H) and NADP(H) is more complicated than for those binding CoA as the pantetheine group of an acyl-CoA is flexible and probably unrecognised by the protein. Consequently, its thioester may be tethered 15 Å away from the pivot point of the β-phosphate of the bound ADP moiety (Fig. 2D). Furthermore, as the reactive distance previously observed for the transfer of an acyl moiety from a surface cysteine residue to a protein lysine is ~ 10 Å^14^, lysine residues that can react with the thioester may be up to ~ 10 Å further away. This creates uncertainty about the location of the thioester and the lysine residues it can react with. Even so, using the complete dataset indicated that lysine residues with higher *N-*acetylation stoichiometry (> 0.4%, n = 46) were also significantly more likely to be near the ribose 5′ carbon atom of the bound nucleotide analogue than less *N-*acetylated residues (< 0.4%, n = 717; Fig. 2F and Supplementary Table S1).

### Lysine *N*-malonylation of glutamate dehydrogenase is decreased by nucleotides

Although the single-subunit data in Supplementary Table S1 supports the hypothesis that nucleotide-binding sites on the surface of proteins enhance *N-*acylation of nearby lysine residues, it cannot fully capture the nucleotide-protein interaction. This is because native proteins are frequently multimeric, some nucleotide-binding sites may be unoccupied in the chosen structure, protein surface features can sterically prevent an acyl-transfer reaction at distances beyond ~ 15 Å^14^, and other mechanisms, such as exposed cysteine residues, can catalyse lysine *N-*acylation on the surface of proteins (Fig. 1B)^14^. Therefore, it was necessary to confirm whether this potential mechanism of *N-*acetylation occurred at particular sites on an individual candidate protein.

**Figure 3:**
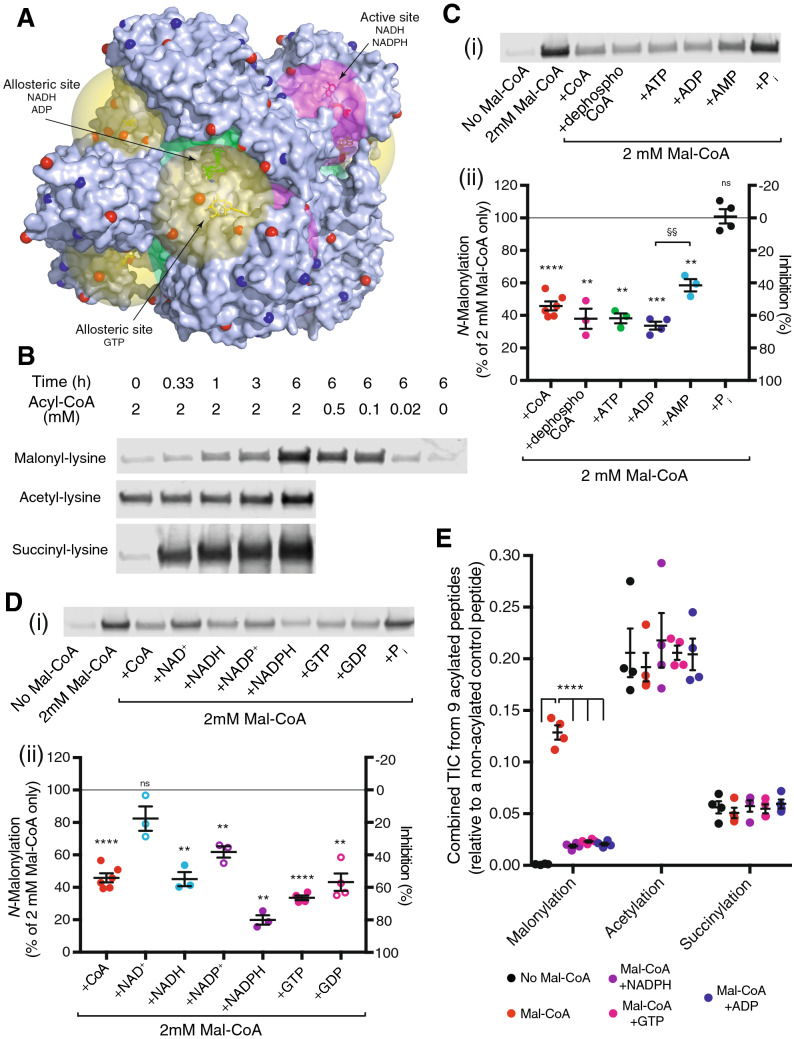
*N-*acetylation of GDH by acyl-CoA is facilitated by nucleotide-binding sites. (**A**) Hexameric GDH (PDB ID: 6DHD) with two bound NADH (pink, active site; green, allosteric site) and one GTP (yellow). A 20 Å sphere centred on the β-phosphate of each bound nucleotide is shown for scale to indicate the reach of the thioester of a bound acyl-CoA. Lysine amines identified as *N-*acetylated in mouse liver^5^ are depicted as red spheres whereas others are blue. (**B**) *N-*acylation of GDH increases with time and acyl-CoA concentration. Purified bovine GDH was exposed to 0–2 mM acetyl-CoA, malonyl-CoA or succinyl-CoA for 0–6 h at 37 °C before resolution on separate SDS-PAGE gels and detection with antibodies specific for anti-acetyllysine, anti-malonyllysine or anti-succinyllysine^7^, respectively. Full-length western blots are in Supplementary Fig. S1. (**C**,**D**) *N-*malonylation can be blocked by excess purine nucleotides. Purified bovine GDH was exposed to 2 mM malonyl-CoA and either 10 mM CoA, dephosphoCoA, ATP, ADP, AMP, GTP, GDP, NADH, NAD^+^, NADPH, NADP^+^ or P_i_ for 6 h at 37 °C before resolution on separate SDS-PAGE gels and detection with an anti-malonyllysine antibody. Full-length western blots are in Supplementary Fig. S1. Data are expressed relative to GDH incubated with 2 mM malonyl-CoA alone from the same gel. Data are the mean ± SEM (n = 3–6). Significance was determined using a paired two-tailed t-test relative to 2 mM malonyl-CoA alone (*) or 2 mM malonyl-CoA with 10 mM ADP (§) signal from the same western blot; ** or §§, p < 0.01; ***, p < 0.001; ****, p < 0.0001. (**E**) *N-*malonylation can be blocked by excess purine nucleotide cofactors. Purified bovine GDH was exposed to 2 mM malonyl-CoA and 10 mM ADP, GTP, or NADPH for 6 h at 37 °C before trypsinisation and LC–MS/MS analysis. The total ion currents (TICs) for 9 *N-*acylated peptides were combined and expressed relative to a control peptide (DDGSWEVIEGR). Data are the mean ± SEM (n = 4). Significance was determined for *N-*malonylation using a one-way ANOVA relative to 2 mM malonyl-CoA alone; ****, p < 0.0001.

Glutamate dehydrogenase (GDH) is an excellent candidate because it contains numerous *N-*acetylation sites and no surface cysteine residues where > 2 Å^2^ of the thiol sulphur atom is solvent exposed (Supplementary Table S1). Furthermore, purified bovine GDH was commercially obtainable and molecular structures of hexameric bovine GDH with NAD(P)H, GTP and ADP bound were available facilitating distance calculations for each of its three nucleotide binding sites (Fig. 3A)^24,25^. Trial incubations indicated that purified bovine liver GDH could be *N-*acetylated, *N-*succinylated and *N-*malonylated in vitro by their respective acyl-CoAs (Fig. 3B)*.* However, purified bovine liver GDH was already extensively *N-*acetylated, resulting in an unsuitably high background for in vitro measurements (Fig. 3B). While succinyl-CoA caused rapid *N-*succinylation, this may be less dependent on nucleotide-binding sites as its reaction mechanism can proceed via succinic anhydride^26^. In contrast, malonyl-CoA is too small to form an anhydride intermediate^26^ and purified mitochondrial GDH was not extensively *N-*malonylated (Fig. 3B). Consequently, malonyl-CoA was chosen to explore whether *N-*acylation of GDH is enhanced by its nucleotide-binding sites.

In vitro, malonyl-CoA caused *N-*malonylation of GDH in a time and concentration dependent manner (Fig. 3B). As phosphate (50 mM) may impact on nucleotides binding to GDH this was omitted from these and future experiments^27^. *N-*malonylation of GDH could be inhibited by a five-fold excess of CoA, dephosphoCoA, ADP or AMP, but not of phosphate (Fig. 3C). Of these ADP was the most effective, preventing 66 ± 2% of the *N-*malonylation. Consistent with the two phosphates of ADP being important for binding to a Rossmann fold^21^, AMP was significantly less effective than ADP, preventing only 41 ± 4% of *N-*malonylation. Hexameric molecular structures for bovine GDH (PDB IDs: 6DHD, 6DHQ and 6DHK) show a catalytic binding site for NAD(P)H and separate allosteric binding sites for GTP and NADH/ADP (Fig. 3A)^24,25^. GTP, GDP, ATP and ADP all prevented ~ 60% of the malonylation (Fig. 3D). Interestingly, NAD^+^, NADH, NADP^+^ and NADPH displayed different propensities to prevent *N-*malonylation with NADPH (80 ± 3%) more inhibitory than NADH (55 ± 4%), NADP^+^ (38 ± 3%), or NAD^+^ (18 ± 8%). This likely reflects cumulative differences in *K*_*D*_ at the three nucleotide-binding sites for each of these competing nucleotides.

When analysed by LC–MS/MS, purified bovine liver GDH contained several *N-*acetylated and *N-*succinylated peptides (Fig. 3E), consistent with its in vivo exposure to acetyl-CoA and succinyl-CoA in the mitochondrial matrix. In contrast, there was little evidence of *N-*malonylation of purified GDH, consistent with the predominant cytosolic location of malonyl-CoA (Fig. 3E). When purified GDH was exposed to 2 mM malonyl-CoA for 6 h at 37 °C there was a 138 ± 30-fold increase in total ion current (TICs) from the nine *N-*malonylated peptides that could be identified with no increase in *N-*acetylated or *N-*succinylated peptides (Fig. 3E). Consistent with quantification by western blot (Fig. 2B,C) addition of 10 mM NADPH, GTP or ADP prevented much of the *N-*malonylation by 2 mM malonyl-CoA (Fig. 3E). Thus, GDH can be *N-*acylated by acyl-CoAs in a manner consistent with their prebinding to cofactor binding-sites.

### Lysine *N*-malonylation of GDH is increased near nucleotide-binding sites

**Figure 4:**
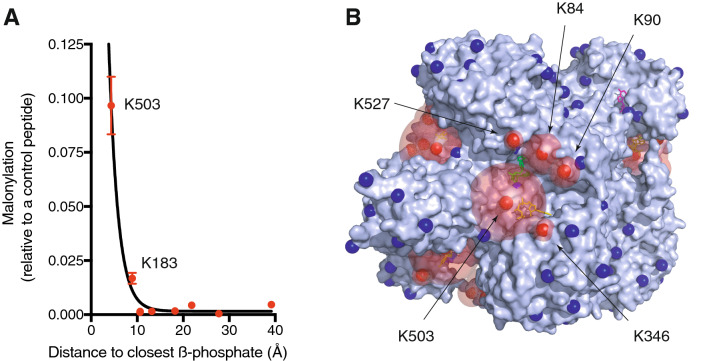
Lysine *N*-acylation of GDH is increased near its nucleotide-binding sites both in vitro and in vivo. (**A**) The *N-*malonylation signal is highest for lysine residues near nucleotide binding sites. GDH was exposed to 2 mM malonyl-CoA for 6 h at 37 °C before trypsinisation and LC–MS/MS. Total ion currents (TICs) for individual *N-*malonylated peptides were expressed relative to a control peptide (DDGSWEVIEGR). Relative TICs are not quantitative as MS response differs between peptides. Distances are from the lysine amine to the β-phosphate of the nearest nucleotide in Supplementary Table S2. Data are the mean ± SEM (n = 4). (**B**) Location and stoichiometry of in vivo lysine *N-*acetylation sites on hexameric GDH (PDB ID: 6DHD). A sphere proportional to *N-*acetylation stoichiometry (1.55 Å/0.2% acetylation stoichiometry) in mouse liver is centred on each lysine amine (NZ). *N*-acetylation stoichiometry is from Weinert et al.^5^. A threshold stoichiometry of > 0.2% is needed to exceed the 1.55 Å van der Vaals radius of the nitrogen atom. Lysine amines not detected as *N-*acetylated or with a stoichiometry of < 0.2% in mouse liver are coloured blue (Supplementary Table S2)^5^.

One prediction of our model is that *N-*acylation of lysine residues will be greater near the nucleotide binding sites of GDH. The majority (88%) of the TICs from the nine *N-*malonylated GDH peptides arise from peptides containing K503 (75 ± 2%; 4.3 Å) and K183 (13 ± 1%; 8.9 Å), the two lysines closest to the β-phosphate of the nucleotide bound to each binding site (Fig. 4A). K503 also accounts for 62 ± 4% and 59 ± 3% of the TICs from pre-existing *N-*acetylation and *N-*succinylation on purified GDH. This suggests that the association of acyl-CoAs with nucleotide-binding sites is not a feature of specific acyl-CoAs and is a significant mechanism by which GDH becomes *N-*acylated in vivo (Supplementary Table S2).

While TICs can only give an approximation of the relative stoichiometry when comparing different peptides, *N-*acetylation stoichiometry for particular lysine residues was previously quantified for mouse liver proteins in vivo, and this showed that K503 was also the most *N-*acetylated lysine residue within GDH (1.63%, Supplementary Table S2)^5^. Furthermore, the five most *N-*acetylated lysine residues of GDH in mouse liver in vivo^5^ cluster near the overlapping allosteric binding sites for GTP and ADP/NADH (Fig. 4B). Interestingly, despite strong *N-*malonylation of K183 near the active site in vitro, this was not observed for either *N-*acetylation or *N-*succinylation with purified GDH or for *N-*acetylation in mouse liver in vivo (Supplementary Table S2)^5^. This likely reflects the presence of competing nucleotides in vivo and binding site-specific differences in *K*_*D*_ between acyl-CoAs and these competing nucleotides.

### Nucleotides decrease malonylation of GDH near their nucleotide-binding sites

**Figure 5:**
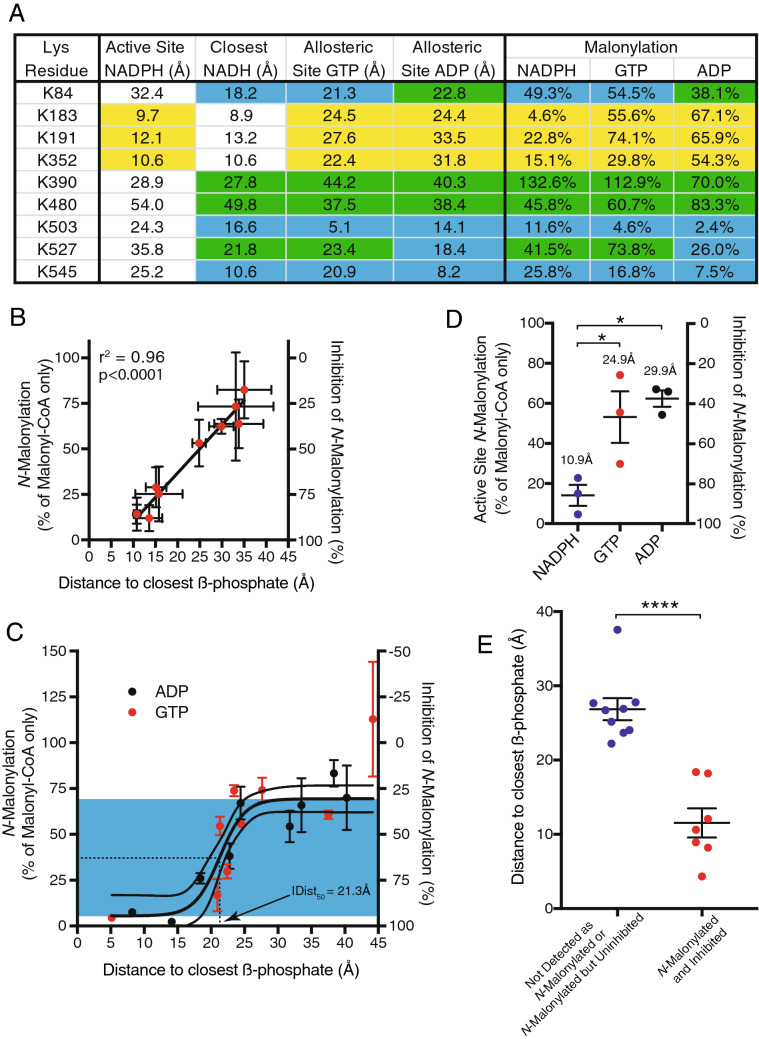
Lysine *N*-malonylation is inhibited by excess NADPH, GTP and ADP near their respective nucleotide-binding sites. Purified bovine GDH was exposed to 2 mM malonyl-CoA and either 10 mM NADPH, GTP or ADP for 6 h at 37 °C. Total ion currents (TICs) for each *N-*malonylated peptide were normalised to a control peptide (DDGSWEVIEGR). Inhibition data are expressed relative to 2 mM malonyl-CoA alone. (**A**) *N-*Malonylation of GDH was inhibited by excess NADPH, GTP or ADP. Distances from the β-phosphate of the inhibitory nucleotide to each lysine amine are shown (see Supplementary Table S2). Residues were grouped by three closest to the active site (yellow; K183, K191 and K352), three closest to the appropriate allosteric site (blue; K503, K545 and K84/K527) and three distal to both sites (green; K390, K480 and K84/K527). Inhibition data are the mean ± SEM (n = 4). (**B**) Inhibition of *N-*malonylation by excess NADPH, GTP or ADP increases near nucleotide binding sites. The mean distance from the β-phosphate of the bound inhibitory nucleotide to the amine of each residue from the groupings in A is shown. Distance data are the mean ± SEM (n = 3); inhibition data are the mean ± SEM (n = 4). p < 0.0001; linear regression. (**C**) Inhibition of *N-*malonylation by excess GTP or ADP increases near their allosteric binding sites. The mean distance from the β-phosphate of the bound inhibitory nucleotide to the amine of each lysine residue is shown. Distance data is the mean ± SEM (n = 3); inhibition data is the mean ± SEM (n = 4). (**D**) *N-*Malonylation of lysine residues (K183, K191 and K352) closest to the active site of GDH is inhibited by excess NADPH, but not GTP or ADP. The mean distance from the β-phosphate of the bound inhibitory nucleotide to the amine of each residue is shown. Data is the mean ± SEM (n = 3); *, p < 0.05; one-way ANOVA. (**E**) Lysine residues not detected as *N-*malonylated or weakly (< 60%) inhibited by excess nucleotides are further from nucleotide binding sites than lysine residues than are *N-*malonylated and strongly (> 60%) inhibited by excess nucleotides. Distances shown are between the β-phosphate of the closest inhibitory nucleotide and the amine of each lysine residue. Significance was determined using a one-way ANOVA; ****, p < 0.0001.

There are three distinct sites on GDH where NAD(P)H, GTP and ADP bind^25^, and adding these nucleotides prevented much of the *N-*malonylation by malonyl-CoA (Fig. 3E). A second prediction of our hypothesis is adding these nucleotides will most effectively inhibit *N-*acylation near their nucleotide-binding sites on GDH. For each inhibitory nucleotide used its β-phosphate position was identified in the appropriate GDH structure (PDB IDs: 6DHD, 6DHQ and 6DHK) and the distance to each lysine residue was calculated (Fig. 5A and Supplementary Table S2). Grouping of lysine residues into the three near the active site (K183, K191 and K352), the three closest to the appropriate allosteric site (K503, K545 and either K84 or K527) and the three farthest from either site (K390, K480 and either K84 or K527), showed a significant correlation between proximity of a lysine to a nucleotide binding site and inhibition of *N-*malonylation by a fivefold excess of a nucleotide with affinity for that site (Fig. 5A,B). Although NADPH is shown binding to the active site of GDH (PDB ID: 6DHQ), it is also likely to bind to the allosteric site containing NADH (Fig. 3D; PDB ID: 6DHD).

Despite the strong correlation in Fig. 5B this relationship is expected to breakdown beyond ~ 25 Å—the combined distance of the ~ 15 Å flexible pantetheine linker of an acyl-CoA when extended (Fig. 2A) and the ~ 10 Å proximity required for the reaction of a thioester with an amine^14^. When distance is graphed relative to the degree of inhibition of *N-*malonylation at that lysine residue, there is a striking sigmoidal relationship for the nucleotides, GTP and ADP, that have only a single binding site (Fig. 5C). For lysine residues < 15 Å from the β-phosphate, the inhibition plateaus at ~ 95%, whereas those further away than 25 Å experience ~ 30% inhibition. Inhibition transitions between these distances, with the distance (IDist_50_) at which a lysine residue experiences half of this inhibition being 22.3 Å (Fig. 5C). In most cases these distances are too far for nucleotides such as GTP or ADP to sterically hinder *N-*malonylation simply by preventing access of malonyl-CoA to the lysine residue.

The probable binding of NADPH to two sites complicated detailed analysis as eight of the nine *N-*malonylated lysine residues were within 25 Å of the β-phosphate of bound NAD(P)H and *N-*malonylation was inhibited > 50% by NADPH at each of these eight lysine residues (Fig. 5A). However, NADPH inhibited *N-*malonylation of residues K183, K191 and K352 near the active site (10.9 Å) more effectively than GTP (24.9 Å) or ADP (29.9 Å) suggesting malonyl-CoA binds to the active site in vitro and *N-*malonylates the proximal lysine residues (Fig. 5D).

### Residues that are neither *N*-malonylated nor inhibited by excess nucleotides are distant from nucleotide-binding sites

A final prediction of our hypothesis is that lysine residues far from the nucleotide-binding sites of GDH will have weak or undetectable *N-*malonylation and that if *N-*malonylation is observed it will be insensitive to inhibition by nucleotides. In addition to the nine malonylated peptides identified here (Fig. 5A), bovine GDH contained a further 22 surface lysine residues that were not identified as *N-*malonylated by LC–MS/MS and where > 2 Å^2^ of their amine NZ atom was solvent exposed (Supplementary Table S2). While this lack of identification could arise because peptides are too short or too long for LC–MS/MS, it could also occur if the degree of *N-*malonylation was low. As peptides identified as *N-*malonylated here ranged from 9 to 20 amino acids in length detection of a further seven of the 22 non-malonylated surface lysine residues might be expected if they were *N-*malonylated (Supplementary Table S2). Furthermore, detectable *N-*malonylation that is weakly inhibited by excess nucleotides could also occur at lysine residues distant from nucleotide-binding sites. Thus, lysine residues that were *N-*malonylated with weak inhibition (< 60%) by all of the excess nucleotides (K390 and K480) and peptides of detectable length but not detected as *N-*malonylated (K68, K110, K162, K200, K212, K363 and K399) were grouped and compared to lysine residues that were *N-*malonylated with strong inhibition (> 60%) by an excess of at least one nucleotide (K84, K183, K191, K352, K503, K527 and K545). This analysis showed that lysine residues having undetectable or weak *N-*malonylation that was insensitive to inhibition by excess nucleotides were further from nucleotide-binding sites (Fig. 5E; p < 0.0001).

## Discussion

Irreversible protein lysine *N-*acylation by acyl-CoAs can occur directly but slowly^13^. We have shown previously that reversible initial *S-*acylation of a cysteine residue can lead to much greater *N-*acetylation of a nearby lysine residue^7,14^. This is because the protein acts as a scaffold to increase the local thioester concentration near the amine of proximal lysine residues^17^. Here we show that *N-*acylation of both mitochondrial protein and purified GDH is diminished by an excess of purine nucleotide cofactors. This suggests that binding sites for nucleotide cofactors can bind acyl-CoAs and thereby facilitate *N-*acylation of nearby lysine residues. A detailed in vitro case study of GDH showed *N-*malonylation by malonyl-CoA was most detectable at K503, which is near two overlapping allosteric sites that bind GTP or ADP/NAD(P)H, and at K183, which is near the active site (Fig. 3A) that binds NAD(P)H. Addition of an excess of NADPH led to the loss of ~ 95% of the *N-*malonylation at both sites, whereas excess of GTP or ADP led to the loss of up to 95% of the *N-*malonylation at K503. In contrast, *N-*malonylation of residues distant (> ~ 25 Å) from these nucleotide-binding sites was either uninhibitable by excess nucleotides or undetectable by LC–MS/MS (Fig. 5E).

These in vitro observations are only partially replicated in vivo, as although K503 is also the main site of *N-*acetylation in mouse liver and on purified GDH, *N-*acetylation of active site K183 is not observed in vivo^5^. The *K*_*M*_ values of bovine liver GDH for NAD(P)H and NAD(P) are ~ 0.02 and ~ 0.2 mM, respectively^28,29^, indicating high occupancy of the active site in vivo. In contrast, to allow responsiveness to metabolic changes, regulatory allosteric sites would be expected to have lower occupancy at physiological cofactor concentrations in vivo, thereby allowing better access to acyl-CoAs. Thus, the relative lack of active-site K183 *N-*acylation in vivo probably reflects the presence of competing nucleotides perhaps combined with differences in occupancy between the active and allosteric sites.

The relevance of this mechanism for proteins other than GDH will have to be addressed on a case-by-case basis. For example, it has been proposed that *N-*malonylation of glyceraldehyde-3-phosphate dehydrogenase (GAPDH) occurs because malonyl-CoA can occupy its binding site for NAD(H)^30,31^. Five of the six most *N-*acetylated lysine residues in mouse liver lie near this NAD(H) binding site (Table S1)^5^. Interestingly, the dataset we used to generate Table S1 had specifically excluded histones as this protein family contains many common peptides^5^. Histones interact with nucleotides using positively charged lysine and arginine residues and many different *N-*acyl lysine modifications have been identified on histones for which there are no known *N-*acyltransferases. This group includes such esoteric histone modifications as *N-*crotonylation^32^, *N-*2-hydroxyisobutyrylation^33^, *N-*lactylation^34^, *N-*propionylation and *N-*butyrylation^35^. Although each of these may represent a transferase-dependent regulatory signal, histones have evolved to interact with nucleotides and consequently may find it difficult to avoid interacting with acyl-CoAs and thereby becoming acylated too.

Finally, we show here that proteins with nucleotide-binding sites for common metabolic cofactors can potentially act as scaffolds for *N-*acylation. This is similar to a previously described mechanism where proximal cysteine residues catalyse *N-*acylation^7,9,14^. Both mechanisms work by enhancing the local thioester concentration, thereby leading to the targeting of certain lysine residues^17^. While this specificity could lead to regulation, *N-*acetylation stoichiometry appears to be, in most cases at least, too low for simple loss of function effects^5,9^. Furthermore, the vast majority of *N-*acylation sites catalysed by cysteine residues appear to lack a biological purpose as they were significantly less conserved within the genomes of vertebrates^14^. The degree to which the nucleotide-binding site driven acylation described here leads to functional regulation rather than just indicating a chemical side reaction will need further investigation.

## Materials and methods

### Materials

Nucleotide cofactor stocks (40 mM) were prepared in 200 mM HEPES and neutralised (pH ~ 7.8; NaOH) where necessary. The pH was checked with pH strips with the exception of FAD because its colour interfered and neutrality had to be assumed. As small differences in pH could be critical, further additional buffering capacity was included in the reaction. Bovine hearts were from cattle of mixed gender and aged 18–22 months (C Humphreys & Sons Abattoir, Chelmsford, UK). Bovine heart mitochondrial membrane fragments were prepared as described previously and stored at − 80 °C until used^36^. Lyophilised glutamate dehydrogenase from bovine liver (Type III, Sigma) was reconstituted in 100 mM HEPES (pH 7.8; NaOH). Rabbit anti-acetyllysine (9441) antibody was from Cell Signaling Technology (Danvers, USA). Rabbit anti-succinyllysine (PTM-401) and anti-malonyllysine (PTM-901) primary antibodies were from PTM Biolabs (Chicago, USA). Mouse anti-NDUFB8 (ab110242) primary antibody was from Abcam (Cambridge, UK). Anti-mouse and anti-rabbit fluorescent secondary antibodies were from LI-COR Biosciences (Lincoln, USA). All other enzymes and chemicals were from Sigma.

### Protein incubations

Bovine heart mitochondrial membrane fragments (100 µg protein) were incubated in 20 µL of NaP_i_/HEPES buffer (50 mM NaH_2_PO_4_, 100 mM HEPES [pH 7.8, NaOH]). GDH (5 µg) was incubated in 20 µL of HEPES buffer (100 mM HEPES [pH 7.8, NaOH]). Incubations were supplemented with 10 mM CoA, dephospho-CoA, pantetheine, ATP, ADP, AMP, GTP, GDP, NADH, NADPH, NAD^+^, NADP^+^_,_ FAD or P_i_ as indicated. The reaction was started with 2 mM of either acetyl-CoA, succinyl-CoA or malonyl-CoA and then incubated for 6 h at 37 °C.

### SDS-PAGE

DTT (5 µL, 1 M) was added to each 20 µL sample of GDH to remove cysteine-bound acetyl groups before mixing 1:1 with SDS-PAGE loading buffer. Samples were separated on a 12% SDS-PAGE gel and samples with no acyl-CoA, 2 mM acyl-CoA, and 2 mM acyl-CoA with 10 mM CoA, were included on all gels. Proteins were transferred from gels to PVDF and incubated overnight at 4 °C with Odyssey Blocking Buffer. The PVDF membrane was probed with 1/2000 dilution of rabbit anti-acetyllysine, rabbit anti-succinyllysine or rabbit anti-malonyllysine and 1/5000 mouse anti-NDUFB8 in Odyssey Blocking Buffer for 1 h at 25 °C. It was washed three times with PBS/0.1% Tween-20 (PBST) before incubation for 1 h at 25 °C with 1/10,000 anti-mouse and anti-rabbit fluorescent secondary antibodies in Odyssey Blocking Buffer. After washing three times with PBST and twice PBS, fluorescence intensity was measured at 680 nm and 800 nm using a LI-COR Odyssey CLx near-infrared imaging system and Image Studio v4.0. Background intensity from a sample with no acyl-CoA was subtracted from the intensity of all acyl-CoA treated lanes and then the data were expressed relative to the intensity of a lane treated with 2 mM acyl-CoA alone.

### Mass spectrometry of GDH

Each 20 µL sample of GDH was incubated with excess DTT (2.5 µL, 50 mM) and SDS (2.5 µL, 10%) for 15 min at 37 °C. This was followed by incubation for 30 min at 37 °C with a greater excess of iodoacetamide (5 µL, 200 mM). After adding DTT (7.5 µL, 1 M), samples were mixed 1:1 with SDS-PAGE loading buffer. Samples were resolved on a 12% SDS-PAGE gel, stained with colloidal Coomassie and the GDH band excised. Proteins contained in the gel bands were cleaved in-gel with trypsin (12.5 mg/µL, overnight at 37 °C). The LC–MS/MS analysis of tryptic peptides was carried out using a Q-Exactive^+^ mass spectrometer (Thermo Scientific) after chromatography on a nanoscale reverse-phase PepMap column (50 µm inner diameter, 150 mm length) with a gradient of 0–40% buffer B (95% acetonitrile, 0.1% formic acid) in buffer A (5% acetonitrile, 0.1% formic acid) over 84 min at 300 nL/min. Four replicate samples were processed for each experimental condition. Peptides were identified using Proteome Discoverer (Thermo Scientific) and the Mascot protein identification program (Matrix Science). All experiments used the mammalian protein subset of the UniProt database (2015_7). Searches were performed using a 5 ppm and 0.01 Da mass tolerance for precursor and fragment ions respectively, while requiring each peptide’s amino/carboxy (N/C) terminus to have trypsin protease specificity and allowing up to two missed tryptic cleavages. *N-*malonylation (+ 87.042 Da), *N-*acetylation (+ 42.037 Da) and *N-*succinylation (+ 100.073 Da) of lysine residues as well as methionine oxidation (+ 15.995 Da) were set as variable modifications and carbamidomethyl (+ 57.051 Da) was used as a fixed modification for cysteines. As sequence information from MS2 data was not present for all peptides in all replicate samples, the raw files of samples lacking appropriate MS2 data were manually analysed and peaks were identified on the basis of their *m/*z values and observed retention time from other replicates. To control for loading differences between runs, ion counts of *N-*acylated peptides were normalised to ion counts of a control peptide DDGSWEVIEGR. This was the only peptide of equivalent size (9–20 residues) with flanking arginine residues and no oxidisable methionines.

### Structural models of CoA analogue-bound mouse proteins

A list of analogues of CoA, ADP, NAD^+^ and FAD that are found as ligands in molecular structures (Table S1A) was created using the ‘similar’ function of the ligand database of the Protein Data Bank (PDB)^37^. A list of *N-*acetylated proteins from mouse liver tissue was taken from published data^5^. If an analogue-bound structural file for an *N-*acetylated protein could be identified, the corresponding mouse protein sequences were taken from UniProt and individually aligned using MODELLER^38^ with those sequences in a non-redundant PDB sequence database clustered at 95%. The best match having a minimum sequence identity of 50% across their entire length of the query sequence and containing the analogue was then used as a structural template. Then the query sequence was structurally aligned with the template and this was used to create five single-subunit predicted structures, with the one with the lowest DOPE score retained for analysis. After alignment, the nucleotide analogue was copied to the modelled mouse structure. Atoms corresponding to the thiol sulphur atom (S1P), the β-phosphate (P2A), α-phosphate (P1A) and the ribose 5′ carbon (C5B) of acetyl-CoA were identified for each nucleotide analogue (Table S1B). Distances between modelled mouse lysine amine atoms (NZ) and bound nucleotide analogue atoms of interest were calculated using trigonometry from the 3D coordinates in the generated PDB files. For each structure the solvent accessible area in Å^2^ of every atom was calculated using areaimol from the CCP4 software suite^39^.

For hexameric bovine GDH, distances between lysine amine atoms (NZ) and bound nucleotide analogue atoms of interest were calculated using trigonometry from the 3D coordinates in published PDB files (6DHD, 6DHQ and 6DHK)^25^. Structures were visualized with the PyMOL Molecular Graphics System (version 1.8.4 Schrödinger, LLC).

### Statistics and data processing

Statistical significance was calculated using Prism version 7 for Mac (GraphPad). Significance for cumulative frequency was determined with a Kaplan–Meier plot followed by a Gehan-Breslow-Wilcoxon test. Otherwise statistical significance was determined using a two-tailed Student’s t-test, one-way ANOVA followed by Sidak’s multiple comparison test or linear regression as indicated.

## Supplementary information

Supplementary Figure S1.

Supplementary Table S1.

Supplementary Table S2.

## Data Availability

All data generated or analysed during this study are included in this published article (and its Supplementary Information Tables S1 and S2). Modelled structures used in the current study are available from the corresponding author on reasonable request.
