# Supplementary material for: Nucleotide-binding sites can enhance N-acylation of nearby protein lysine residues
Source: Sci Rep. 2020 Nov 20;10:20254. doi: 10.1038/s41598-020-77261-1 (PMC7680127; doi:10.1038/s41598-020-77261-1)
Supplement: Supplementary file 1 — Supplementary Figure S1. [file 41598_2020_77261_MOESM1_ESM.docx]

**Supplementary Information**

**Nucleotide-binding sites can enhance *N*-acylation of nearby protein lysine residues**

Andrew M. James^1,4*^, Anthony C. Smith^1^, Shujing Ding^1^, Jack W. Houghton^2^, Alan J. Robinson^1^, Robin Antrobus^2^, Ian M. Fearnley^1^, and Michael P. Murphy^1,3,*^

^1^Medical Research Council Mitochondrial Biology Unit, University of Cambridge, Cambridge, CB2 0XY, UK

^2^Cambridge Institute of Medical Research, University of Cambridge, Cambridge, CB2 0XY, UK

^3^Department of Medicine, University of Cambridge, Cambridge, CB2 0QQ, UK

^4^Lead contact

^*^Correspondence: aj@mrc-mbu.cam.ac.uk; mpm@mrc-mbu.cam.ac.uk

**Supplementary Figure S1** Full length western blots for Fig. 3. (**A-C**) *N-*acylation of GDH increases with time and acyl-CoA concentration. Purified bovine GDH was exposed to 0-2 mM acetyl-CoA (A), succinyl-CoA (B) or malonyl-CoA (C) for 0-6 h at 37˚C before resolution on separate SDS-PAGE gels and detection by western blot with anti-acetyllysine (A), anti-succinyllysine (B) or anti-malonyllysine (C) antibodies, respectively. (**D-E**) *N-*malonylation can be blocked by excess purine nucleotides. Purified bovine GDH was exposed to 2 mM malonyl-CoA and either 10 mM CoA, dephosphoCoA, ATP, ADP, AMP, GTP, GDP, NADH, NAD^+^, NADPH, NADP^+^, P_i_ or pantetheine for 6 h at 37˚C before resolution on separate SDS-PAGE gels and detection by western blot with an anti-malonyllysine antibody. Molecular weight (MW) was determined using Precision Plus Protein^TM^ Dual color standards (Biorad).
